# Assessing the Role of Ultrasound Scanning in Improving Pregnancy Outcomes in Potiskum and Neighboring Rural Communities in Yobe State, Nigeria

**DOI:** 10.7759/cureus.79768

**Published:** 2025-02-27

**Authors:** Olajide J Olagunju, Ben Egbo, Olagoke O Osanyinlusi, Olayinka E Olagunju, Seyi E Olorunmolu

**Affiliations:** 1 Division of Infectious Disease, Case Western Reserve University School of Medicine, Cleveland, USA; 2 Department of Radiology, Potiskum Medical Center, Potiskum, NGA; 3 Department of Anesthesiology, Washington University School of Medicine in St. Louis, St. Louis, USA; 4 Department of Zoology, Obafemi Awolowo University, Ile-Ife, NGA; 5 Department of Anatomy, University of Ilorin, Ilorin, NGA

**Keywords:** antenatal care visits, northeastern nigeria, pregnancy outcome, role of ultrasound imaging, rural and underserved communities

## Abstract

Background

Accurate pregnancy dating is vital for timely prenatal interventions that reduce maternal and neonatal risks. In underserved areas such as Potiskum, Yobe State, Nigeria, limited awareness, poor healthcare access, and cultural barriers hinder early pregnancy detection, gestational age estimation, and monitoring. While ultrasound, the gold standard for dating pregnancies, could address these challenges, its impact in these regions remains underexplored.

Methods

This retrospective cross-sectional study analyzed five years of data (2019-2023) from the electronic health records (EHRs) of Potiskum Medical Center. The study included 15,862 records of women who resided within a 20-km radius of Potiskum and underwent ultrasound scanning during pregnancy. Descriptive statistics were carried out to understand the characteristics of the study population, and logistic regression models were used to evaluate associations between pregnancy outcomes and factors including ultrasound scan frequency, maternal age, educational attainment, socioeconomic status, parity, and marital status.

Results

The study revealed that women who underwent a first-trimester ultrasound with one or two additional scans had significantly higher odds of improved pregnancy outcomes (adjusted odds ratio (AOR) = 2.26, 95% confidence interval (CI) = 1.22-2.46, and AOR = 3.84, 95% CI = 2.34-4.58, respectively, p < 0.0001). In contrast, third-trimester-only scans had lower odds of favorable outcomes (AOR = 0.42, 95% CI = 0.31-0.52, p = 0.001). Maternal age was also a significant factor, with younger women (21-25 years) demonstrating the highest odds of favorable outcomes (AOR = 4.24, 95% CI = 3.28-4.86, p < 0.001). Higher educational attainment and socioeconomic status were positively associated with improved outcomes, with tertiary education (AOR = 2.84, 95% CI = 1.53-5.29, p = 0.001) and high income (AOR = 3.84, 95% CI = 3.23-4.06, p < 0.0001) showing the strongest effects. Moderate parity was also associated with better outcomes, while high parity showed weaker odds (AOR = 0.65, 95% CI = 0.28-0.86, p < 0.0001).

Conclusion

The findings highlight the transformative potential of early and all-trimester ultrasound use in improving pregnancy outcomes in rural communities. Factors such as maternal age, education, socioeconomic status, and parity significantly influence outcomes, emphasizing the need for targeted interventions to promote equitable access to quality antenatal care. Addressing these gaps can improve pregnancy outcomes, contributing to global efforts to reduce maternal and child mortality in resource-limited settings. Further research is needed to explore barriers to ultrasound access and develop strategies to enhance antenatal care utilization in underserved regions.

## Introduction

Accurate ultrasound scanning plays a crucial role in effective prenatal care and better pregnancy outcomes by enabling early detection of complications and facilitating timely interventions that significantly reduce maternal and neonatal morbidity and mortality. However, healthcare challenges in northeastern Nigeria continue to undermine pregnancy outcomes, with maternal mortality exceeding 800 deaths per 100,000 live births [1] and a preterm birth rate of 9.9% [2]. Limited healthcare infrastructure, financial constraints, and cultural barriers prevent many women from accessing essential antenatal care. Also, misconceptions about contraception and inadequate education contribute to high fertility rates and poor maternal health. Evaluating the impact of ultrasound scanning in pregnancy in this region is essential for improving outcomes, as it can enhance clinical decision-making, encourage timely medical interventions, strengthen healthcare services, and expand reproductive health education.

Ultrasound scanning, regarded globally as the gold standard for estimating gestational age, has revolutionized prenatal care by enabling accurate pregnancy dating and early detection of potential complications [3,4]. This technology equips healthcare providers with critical information to make informed maternal and fetal health decisions, thereby improving outcomes [4]. In regions such as Potiskum and rural Yobe State, where traditional pregnancy management methods are still prevalent, introducing ultrasound scanning presents a transformative opportunity to bridge significant gaps in pregnancy management and improve maternal and neonatal outcomes.

A significant gap exists in understanding the direct impact of ultrasound scanning on pregnancy and its subsequent influence on pregnancy outcomes in this region. Despite the global recognition of ultrasound as a transformative tool in maternal healthcare [5,6], its specific role in addressing the unique challenges faced by rural communities in Potiskum and Yobe State remains underexplored. This lack of targeted research underscores an urgent need to investigate how integrating ultrasound scanning into prenatal care can enhance early pregnancy detection, improve gestational age estimation, and optimize maternal and neonatal health outcomes. By filling this critical gap, such research has the potential to inform evidence-based interventions, bridge existing healthcare disparities, and ultimately contribute to reducing maternal and neonatal morbidity and mortality in these underserved communities.

This study explores the impact of ultrasound scanning in addressing these challenges and its role in improving pregnancy outcomes in rural Yobe State, Nigeria. Using five years of data from Potiskum Medical Center, a key provider of ultrasound services in the region, the research evaluates how this technology enhances early pregnancy detection, facilitates the management of high-risk pregnancies, and contributes to reducing maternal and neonatal mortality rates. Ultimately, the study underscores the transformative potential of ultrasound scanning in advancing maternal and neonatal health in Potiskum and rural Yobe State. By addressing knowledge gaps in the role of ultrasound scanning and pregnancy outcomes, this work contributes to the global effort to reduce maternal and child mortality, aligning with international maternal health goals [7]. It serves as a call to action for the adoption of innovative and evidence-based strategies to combat the persistent challenges of maternal and neonatal healthcare in sub-Saharan Africa.

Aim and objective

This study aims to provide actionable insights into how ultrasound scanning can bridge existing gaps in pregnancy outcomes in underserved rural settings in Yobe State, Nigeria.

The objective of this study is to evaluate the impact of ultrasound scanning on pregnancy outcomes in rural settings.

## Materials and methods

Study design

This retrospective cross-sectional study utilized five years of data (2019-2023) from the electronic health records (EHRs) of Potiskum Medical Center, a leading healthcare facility providing ultrasound services to Potiskum and neighboring rural communities in Yobe State, Nigeria.

Study setting

Potiskum Medical Center, the fastest-growing private healthcare facility in the region, serves as a referral center for over 500,000 individuals across Potiskum and rural communities in Yobe State, Nigeria. Although Potiskum has one tertiary health facility, three private hospitals, and several primary healthcare centers, only General Hospital Potiskum and Potiskum Medical Center had fully functioning radiology units during the study period. The facility is well-equipped with ultrasound technology and is a primary hub for antenatal care services in the region. The center's EHR system captures detailed records of patient demographics, clinical presentations, ultrasound findings, and pregnancy outcomes.

Study population

The study included pregnant women who underwent ultrasound scanning at Potiskum Medical Center during the study period (January 1, 2019, to December 31, 2023). Inclusion criteria were pregnant women with recorded ultrasound scans used for gestational age estimation, availability of complete data on pregnancy outcomes (e.g., normal pregnancy outcome, ectopic gestation, abortions, preterm births, low birth weight, and intrauterine fetal death), no known fetal anomalies at the time of ultrasound, and residents of Potiskum or neighboring rural communities within 20-km distance. Exclusion criteria were incomplete medical records or missing key data points, non-pregnancy-related ultrasound records, pregnancies with congenital anomalies, and non-residents of the study area.

Data preparation

The variables considered for this study were closely reviewed, and recoding was conducted for this study to align with existing literature from peer-reviewed journals. The outcome of interest (pregnancy outcome) was recoded as a binary variable to include favorable outcomes and adverse outcomes (ectopic gestation, abortion, preterm delivery, low birth weight, and intrauterine fetal death). The frequency of ultrasound scans during pregnancy was simplified into five levels: first trimester only, first trimester with one other scan, first trimester with two other scans, second trimester only, and third trimester only. Maternal age was recoded from detailed age to the following age categories: 16-20, 21-26, 26-30, 31-35, and above 35 years. Maternal educational status was simplified into four categories: no formal education, primary education, secondary education, and tertiary education. Socioeconomic status was simplified into three levels: low income (<50,000 Naira/month), middle income (50,000-250,000 Naira/month), and high income (>250,000 Naira/month). Marital status was categorized as "married" and "not married." Parity levels were grouped into three levels: low parity (0-2), moderate parity (3-4), and high parity (≥5).

Statistical analysis

A total of 15,862 records were included in this study. Descriptive statistics were applied to understand the characteristics of the study population. Simple logistic regression analyses were carried out to examine the independent association between the explanatory variable, covariates, and the outcome. The backward selection method was employed to include variables for the multivariate logistic regression model, which assessed the combined association between pregnancy outcomes and ultrasound usage frequency, and other covariates. Variables demonstrating statistical significance in the simple logistic regression were included in the multivariate model to evaluate their combined effects. Statistical significance was set at p < 0.05, and all analyses were performed using the Statistical Analysis System (SAS) version 9.4 for Windows (SAS Institute Inc., Cary, North Carolina).

## Results

This study included 15,862 records. An overview of key demographic and clinical characteristics, as shown in Table 1, revealed that regarding ultrasound scan frequency, 32.44% of women had scans in the first trimester only, 17.92% combined a first-trimester scan with one other, 14.11% had a first-trimester scan with two others, 26.82% had a scan in the second trimester only, and 8.71% had scans exclusively in the third trimester. Maternal age distribution showed that the majority were aged 26-30 years (36.90%), followed by 21-25 years (24.60%) and 31-35 years (21.50%), while only 9.40% and 7.60% fell in the 16-20 and >35 age groups, respectively. Educational status revealed that 35.38% of women had secondary education, 26.48% had primary education, 20.34% had no formal education, and 17.80% achieved tertiary education. Socioeconomic status predominantly comprised middle-income participants (59.44%), with 25.92% in low income and 14.64% in high income. Parity distribution showed that 45.47% of women had moderate parity, 32.29% had low parity, and 22.24% had high parity. Marital status indicated that the vast majority (90.61%) were married, while 9.39% were not married.

**Table 1:** Descriptive data statistics for assessing the role of ultrasound scanning in improving pregnancy outcomes in Potiskum and neighboring rural communities in Yobe State, Nigeria

Variables	Categories	Frequency (N=15,862)	Percentage (%)
Ultrasound scan frequency	First trimester only	5,144	32.44
	First trimester with one other scan	2,843	17.92
	First trimester with two other scans	2,240	14.11
	Second trimester only	4,254	26.82
	Third trimester only	1,381	8.71
Maternal age	16-20 years	1,491	9.40
	21-25 years	3,902	24.60
	26-30 years	5,853	36.90
	31-35 years	3,409	21.50
	>35 years	1,207	7.60
Educational status	No formal education	3,226	20.34
	Primary education	4,200	26.48
	Secondary education	5,612	35.38
	Tertiary education	2,824	17.80
Socioeconomic status	Low income	4,111	25.92
	Middle income	9,428	59.44
	High income	2,323	14.64
Parity	Low parity	5,121	32.29
	Moderate parity	7,213	45.47
	High parity	3,528	22.24
Marital status	Married	14,372	90.61
	Not married	1,490	9.39

Bivariate logistic regression analysis was conducted, as shown in Table 2, to evaluate the potential factors influencing the role of ultrasound scanning in pregnancy outcomes in Potiskum and neighboring rural communities in Yobe State, Nigeria. The analysis focused on key factors, including ultrasound scan frequency, maternal age, educational status, socioeconomic status, parity, and marital status. By actively examining these variables, we aimed to identify independent and significant associations between these factors and pregnancy outcomes.

**Table 2:** Bivariate logistic regression to assess the independent effect of potential factors influencing the role of ultrasound scanning in improving pregnancy outcomes in Potiskum and neighboring rural communities in Yobe State, Nigeria OR: odds ratio, CI: confidence interval

Variables	Categories	OR (95% CI)	P value
Ultrasound scan frequency	First trimester only	Reference	
	First trimester with one other scan	2.84 (1.42-2.86)	<0.0001
	First trimester with two other scans	4.26 (2.48-4.62)	<0.0001
	Second trimester only	1.82 (1.22-2.23)	<0.0001
	Third trimester only	0.49 (0.42-0.56)	<0.0001
Maternal age	16-20 years	Reference	
	21-25 years	6.22 (1.44-6.92)	<0.001
	26-30 years	5.88 (1.46-6.54)	<0.001
	31-35 years	0.44 (0.42-0.56)	<0.001
	>35 years	0.38 (0.38-0.52)	<0.001
Educational status	No education	Reference	
	Primary education	1.02 (1.01-1.12)	0.002
	Secondary education	1.48 (1.21-1.80)	<0.0001
	Tertiary education	2.66 (2.43-3.10)	0.003
Socioeconomic status	Low income	Reference	
	Middle income	2.48 (1.36-3.43)	<0.0001
	High income	4.22 (3.62-4.82)	<0.0001
Parity	Low parity	Reference	
	Moderate parity	1.24 (1.11-1.38)	<0.0001
	High parity	0.72 (0.44-0.82)	<0.0001
Marital status	Married	Reference	
	Not married	0.83 (0.80-1.92)	0.6713

The multivariate analysis reveals significant associations between various factors and the role of ultrasound scanning in improving pregnancy outcomes in Potiskum and neighboring rural communities, as shown in Table 3. Regarding ultrasound scan frequency, having a first-trimester scan with one or two additional scans significantly increased the odds of favorable outcomes, with the strongest association observed for two additional scans (adjusted odds ratio (AOR) = 3.84, 95% CI = 2.34-4.58, p < 0.0001). In contrast, scans conducted only in the third trimester were associated with lower odds of favorable outcomes (AOR = 0.42, 95% CI = 0.31-0.52, p = 0.001). Maternal age also played a crucial role, with younger mothers (21-30 years) showing the highest likelihood of favorable outcomes, particularly those aged 21-25 (AOR = 4.24, 95% CI = 3.28-4.86, p < 0.001), while older mothers (>35 years) had reduced odds (AOR = 0.32, 95% CI = 0.24-0.48, p < 0.001). Educational attainment showed a strong positive influence, with the highest odds of favorable outcomes observed among mothers with tertiary education (AOR = 2.84, 95% CI = 1.53-5.29, p = 0.001), followed by those with secondary education (AOR = 1.60, 95% CI = 1.04-2.06, p < 0.0001). Socioeconomic status further highlighted disparities, as middle-income mothers (AOR = 2.22, 95% CI = 1.13-3.11, p < 0.0001) and high-income mothers (AOR = 3.84, 95% CI = 3.23-4.06, p < 0.0001) were significantly more likely to experience favorable outcomes compared to those in the low-income group. Parity also showed a notable effect, with moderate parity having the strongest association with favorable outcomes (AOR = 1.18, 95% CI = 1.72-2.10, p < 0.0001), while high parity showed weaker odds (AOR = 0.65, 95% CI = 0.28-0.86, p < 0.0001).

**Table 3:** Multivariate logistic regression to assess the combined effects of factors influencing the role of ultrasound scanning in improving pregnancy outcomes in Potiskum and neighboring rural communities in Yobe State, Nigeria AOR: adjusted odds ratio, CI: confidence interval

Variables	Categories	AOR (95% CI)	P value
Ultrasound scan frequency	First trimester only	Reference	
	First trimester with one other scan	2.26 (1.22-2.46)	<0.0001
	First trimester with two other scans	3.84 (2.34-4.58)	<0.0001
	Second trimester only	1.21 (1.10-1.33)	<0.0001
	Third trimester only	0.42 (0.31-0.52)	0.001
Maternal age	16-20 years	Reference	
	21-25 years	4.24 (3.28-4.86)	<0.001
	26-30 years	3.86 (1.58-9.42)	0.003
	31-35 years	0.39 (0.38-0.52)	0.002
	>35 years	0.32 (0.24-0.48)	<0.001
Educational status	No education	Reference	
	Primary education	1.21 (1.08-1.36)	0.001
	Secondary education	1.60 (1.04-2.06)	<0.0001
	Tertiary education	2.84 (1.53-5.29)	0.001
Socioeconomic status	Low income	Reference	
	Middle income	2.22 (1.13-3.11)	<0.0001
	High income	3.84 (3.23-4.06)	<0.0001
Parity	Low parity	Reference	
	Moderate parity	1.18 (1.12-2.10)	<0.0001
	High parity	0.65 (0.28-0.86)	<0.0001

## Discussion

This study evaluated the role of ultrasound scanning in improving pregnancy outcomes in Potiskum and neighboring rural communities in Yobe State, Nigeria, by examining key maternal factors. The findings demonstrate that the frequency and timing of ultrasound scans, maternal age, educational status, socioeconomic status, and parity significantly influence pregnancy outcomes.

Despite the World Health Organization (WHO) recommendation of at least one ultrasound scan before 24 weeks gestation, the frequency and timing of ultrasound scans emerged as critical determinants of improved outcomes. Women who underwent a first-trimester scan with one or two additional scans in the two other trimesters had significantly higher odds of favorable pregnancy outcomes compared to those who had only a first-trimester scan. This is consistent with existing evidence highlighting the importance of early and repeated ultrasounds in all trimesters of pregnancy in monitoring growth and addressing complications early. Yousefpour Shahrivar et al., in their study of ultrasound imaging in pregnancy, emphasize that conducting ultrasound imaging at different trimesters of pregnancy plays a vital role in monitoring fetal well-being and significantly improving pregnancy outcomes [8]. Repeated ultrasound scans in each trimester allow healthcare providers to track fetal development, detect potential complications early, and implement timely interventions [9-11]. This continuous assessment enhances clinical decision-making, reduces maternal and neonatal risks, and ultimately contributes to safer pregnancies and better health outcomes.

However, women who underwent scans exclusively in the third trimester had significantly lower odds of favorable pregnancy outcomes, underscoring the limited impact of late ultrasound scans when complications or anomalies might already be advanced. This is consistent with existing studies that have found little or no sensitivity of third-trimester ultrasound imaging to adverse pregnancy outcomes. In their study comparing two (32/38 weeks) versus one (36 weeks) ultrasound protocol for the detection of decreased fetal growth and adverse perinatal outcomes, Nieto-Tous et al. agreed that third-trimester ultrasound has low sensitivity to adverse perinatal outcomes [12]. Also, Al-Hafez et al. concluded that third-trimester ultrasounds do not decrease the rate of perinatal outcomes [13]. These findings highlight the need for education and accessibility initiatives to encourage early and consistent antenatal ultrasound use.

Maternal age also played a significant role in pregnancy outcomes, with younger women (21-30 years) demonstrating the highest odds of favorable pregnancy outcomes compared to those within 16-20 years. This is likely due to better physiological fitness for pregnancy and fewer underlying health conditions in younger women compared to older women, particularly those over 35 years, who had significantly lower odds of favorable pregnancy outcomes. These findings align with global studies showing advanced maternal age as a risk factor for pregnancy complications and adverse pregnancy outcomes. In his study of advanced maternal age and adverse pregnancy outcomes, Frick submitted that advanced maternal age is associated with a wide range of risk for adverse pregnancy outcomes [14]. In a national study in Denmark, Frederiksen et al. concluded that while multiple factors contribute to adverse pregnancy outcomes, advanced maternal age remains a major determinant of the overall risk [15]. Multiple other studies have consistently identified advanced maternal age as a significant risk factor for adverse pregnancy outcomes [16-20]. These studies emphasize the importance of enhanced prenatal monitoring and early interventions to mitigate risks associated with delayed childbearing. By recognizing advanced maternal age as a key contributor to adverse pregnancy outcomes, healthcare providers can implement targeted strategies to improve maternal and neonatal health outcomes. Programs targeting older mothers may need to integrate closer ultrasound scan monitoring and personalized interventions to address these risks.

Educational attainment showed a strong positive association with improved outcomes, with the highest odds observed among women with tertiary education compared to those with no formal education. This finding is consistent with the existing literature on the association between maternal education level and pregnancy outcomes. Rogne et al. stated in their study that lower maternal education levels increase the risk of pregnancy complications [21]. Also, a study carried out in Ethiopia by Ketema et al. concluded that pregnant women with higher education levels demonstrated better preparedness for childbirth and potential complications compared to those with no formal education [22]. This finding underscores the critical role of maternal education in accessing and utilizing antenatal care services, including ultrasounds, and adhering to health-promoting behaviors during pregnancy. Women with higher education are more likely to understand the importance of early interventions and effectively navigate healthcare systems. In contrast, women with no education, who served as the reference group, had the poorest outcomes, highlighting the urgent need to address educational disparities in this region.

The socioeconomic status of women in this region also significantly influenced pregnancy outcomes. Women from middle- and high-income groups had markedly better odds of favorable outcomes than their low-income counterparts. This is also consistent with documented evidence in existing literature. A study by Keenan-Devlin et al. concluded that maternal socioeconomic status is a predictor of adverse pregnancy outcomes [23]. Another study conducted in India documented that individuals from lower socioeconomic backgrounds often struggle to access healthcare due to limited awareness and inadequate healthcare resources, resulting in a higher risk of adverse pregnancy outcomes [24]. Multiple other studies also documented the effect of socioeconomic status on pregnancy outcomes [25-29]. This reflects disparities in access to healthcare resources, including the ability to afford ultrasound scans and address complications promptly. These findings reinforce the importance of targeted policies to subsidize antenatal care costs for low-income populations and improve the availability of ultrasound services in underserved rural communities.

Parity was another significant factor, with moderate parity associated with the highest odds of improved outcomes. Women with low parity also showed favorable outcomes, but high parity was associated with poorer outcomes. This finding aligns with previous studies that have established a strong link between parity and pregnancy outcomes. In their study on parity and pregnancy outcomes, Bai et al. emphasized the importance of prioritizing care for women who have given birth four or more times to mitigate potential risks [29]. Also, a large multicenter study concluded that high parity is associated with adverse perinatal outcomes [30]. This pattern may be due to the cumulative physiological strain associated with multiple pregnancies, limited access to family planning resources, and adequate antenatal care, including timely ultrasound imaging in rural settings. These findings emphasize the need for family planning programs and antenatal care interventions to address the risks associated with high parity.

Overall, the findings of this study underscore the importance of early and all-trimester ultrasound scanning, improving maternal education, addressing socioeconomic disparities, and tailoring interventions to maternal age and parity. Policymakers and healthcare providers should focus on promoting equitable access to high-quality antenatal care services, particularly for low-income and less-educated women. By addressing these factors, maternal and fetal health outcomes in rural communities such as those in Yobe State, Nigeria, can be significantly improved. Future studies should further explore the barriers to accessing early ultrasound scans and investigate additional interventions to enhance antenatal care utilization in resource-limited settings.

Strengths and limitations

This study has several strengths that enhance its significance. With a large sample size of 15,862 records and five years of real-world data from Potiskum Medical Center, the findings are robust and highly relevant to rural settings. It addresses critical gaps in understanding the role of ultrasound in underserved communities in northeastern Nigeria, the first of its kind to the best of our knowledge. Using rigorous statistical methods, it highlights the importance of early and all-trimester ultrasound imaging and offers actionable, policy-relevant strategies for improving antenatal care in resource-limited settings.

However, this study has some limitations as it did not assess maternal health, nutritional status, or environmental factors, which are critical to pregnancy outcomes. Also, unfavorable outcomes were not reported in their different proportion. Additionally, the study is a single-center study, and self-reported socioeconomic status may introduce reporting bias.

## Conclusions

This study underscores the critical role of ultrasound scanning in improving pregnancy outcomes in Potiskum and neighboring rural communities in Yobe State, Nigeria. The findings highlight that early and all-trimester ultrasound scanning significantly enhances pregnancy outcomes, particularly in the first trimester with one or two additional scans in the second and third trimesters. Key maternal factors, including younger age, higher educational attainment, better socioeconomic status, and moderate parity, were also positively associated with favorable outcomes. Conversely, limited or late use of ultrasound, older maternal age, lower educational attainment, and high parity were linked to poorer outcomes.

Ultimately, this study supports the adoption of evidence-based strategies to enhance maternal and neonatal health in resource-limited settings and the WHO protocol of at least one ultrasound scan before 24 weeks gestation. By addressing these challenges, policymakers and healthcare providers can contribute to reducing maternal and neonatal morbidity and mortality in Nigeria, aligning with global maternal health goals. Further research is required to explore barriers to ultrasound access and develop innovative interventions that promote equitable healthcare delivery in rural communities.
