# Correction: Assessing the Role of Ultrasound Scanning in Improving Pregnancy Outcomes in Potiskum and Neighboring Rural Communities in Yobe State, Nigeria

**DOI:** 10.7759/cureus.c441

**Published:** 2026-05-29

**Authors:** Olajide J Olagunju, Ben Egbo, Olagoke O Osanyinlusi, Olayinka E Olagunju, Seyi E Olorunmolu

**Affiliations:** 1 Division of Infectious Disease, Case Western Reserve University School of Medicine, Cleveland, USA; 2 Department of Radiology, Potiskum Medical Center, Potiskum, NGA; 3 Department of Anesthesiology, Washington University School of Medicine in St. Louis, St. Louis, USA; 4 Department of Zoology, Obafemi Awolowo University, Ile-Ife, NGA; 5 Department of Anatomy, University of Ilorin, Ilorin, NGA

This article has been corrected to update numerous confidence interval ranges which were incorrectly entered at the time of submission. No other changes have been made.

